# Silencing c-Myc Enhances the Antitumor Activity of Bufalin by Suppressing the HIF-1α/SDF-1/CXCR4 Pathway in Pancreatic Cancer Cells

**DOI:** 10.3389/fphar.2020.00495

**Published:** 2020-04-17

**Authors:** Xia Liu, Yayun Zhou, Jiamin Peng, Bei Xie, Qiyang Shou, Jianchao Wang

**Affiliations:** ^1^ The Second Clinical Medical College, Zhejiang Chinese Medical University, Hangzhou, China; ^2^ Department of Clinical Laboratory, Tongde Hospital of Zhejiang Province, Hangzhou, China

**Keywords:** pancreatic cancer, c-Myc, bufalin, HIF-1α/SDF-1/CXCR4 pathway, invasion, migration

## Abstract

**Background:**

Pancreatic cancer is one of the most aggressive malignancies. Bufalin, a traditional Chinese medicine, has been used to treat pancreatic cancer as an antitumor agent although the mechanism by which it exerts its effects is still unclear. c-Myc has been found to be overexpressed in more than half of human cancers including pancreatic cancer. However, the role of c-Myc in pancreatic cancer cells and its influence in bufalin-treated pancreatic cancer are yet to be clarified. The present study aimed to investigate the role of c-Myc in the antitumor activity of bufalin in pancreatic cancer.

**Methods:**

c-Myc siRNA and overexpression plasmid were transfected into pancreatic cancer cells to construct the cell models. c-Myc expression was detected *via* quantitative real-time polymerase chain reaction and western blot. The effect of c-Myc on bufalin-induced inhibition of cell proliferation was detected *via* CCK-8 assay. Cell apoptosis and the cell cycle were analyzed *via* flow cytometry. Cell invasion and migration was detected *via* Transwell and wound healing assays, respectively. In addition, the effect of bufalin on the suppression of tumor growth *in vivo* was studied in nude mice model subcutaneously injected with PANC-1 and SW1990 cells. Hematoxylin-eosin and terminal deoxynucleotidyl transferase dUTP nick-end labeling assay were used to evaluate pathological changes *in vivo*. The expression of HIF-1α/SDF-1/CXCR4 were detected *via* western blot.

**Results:**

CCK-8 assay showed that bufalin could inhibit the proliferation of pancreatic cancer cell, and c-Myc downregulation enhanced this effect. Similarly, c-Myc downregulation enhanced the effect of bufalin on cell cycle arrest, apoptosis, and the invasion and migration of pancreatic cancer cell *in vitro*. Further mechanism assay showed that c-Myc enhances the effect by regulating the HIF-1α/SDF-1/CXCR4 signaling pathway. The *in vivo* studies verified the results that c-Myc enhances the effect of bufalin through regulation of the HIF-1α/SDF-1/CXCR4 pathway.

**Conclusions:**

Downregulation of c-Myc enhanced the antitumor activity of bufalin in pancreatic cancer cells by suppressing the HIF-1α/SDF-1/CXCR4 pathway. These findings indicate that c-Myc inhibitors could enhance the clinical therapeutic effect of bufalin and may expand the clinical application of bufalin accordingly.

## Introduction

Pancreatic cancer is the one of the most aggressive digestive system malignancies worldwide, with a 5-year survival rate of less than 5% (Siegel et al., 2015). Due to the insidious onset of pancreatic cancer, the difficulty of early diagnosis, and the high risk of metastasis, it is usually diagnosed at an advanced stage, and the mortality rate has been increasing (Vincent et al., 2011). Surgical resection is an effective treatment modality for pancreatic cancer, but fewer than 20% of patients are eligible for curative resection (Lin et al., 2015). Therefore, gemcitabine-based chemotherapy has been the standard regimen for advanced pancreatic cancer. However, the overall survival of pancreatic cancer patients is extremely poor because of high chemotherapy resistance (Singh et al., 2015). Thus, there is a crucial need for better understanding of the underlying mechanisms of tumorigenesis and more effective chemotherapeutic strategies.

Chansu, a traditional Chinese medicine, is a dried toad venom secreted from the auricular and skin glands of toads and has been widely used as an analgesic, a cardiac drug, an anti-microbial agent, a local anesthetic, and an anti-inflammatory drug (Qi et al., 2014). Some studies have shown that bufalin, one of the major active components of Chansu, induces apoptosis and cell cycle arrest, suppressing the invasion and migration of cancer cells by regulating various signaling pathways and genes, in several cancers such as leukemia, lung cancer, liver cancer, prostate cancer, bladder cancer, and osteosarcoma (Cheng et al., 2019; Lan et al., 2019). As such, bufalin might be a potential antitumor chemotherapeutic drug. However, the potential mechanisms by which bufalin exerts anticancer activity in pancreatic cancer remains to be further investigated.

The mechanism by bufalin exerts its antitumor activity at the molecular level is extremely complex and involves the regulation of various signaling pathways and genes. c-Myc, a proto-oncogene, has been one of the research hotspots in tumor molecular biology since it was first discovered more than 30 years ago as a cellular homolog of v-myc, which induces tumorigenesis in birds (Hsieh et al., 2015; Majello and Perini, 2015). In addition, the c-Myc is aberrantly overexpressed in over half of human cancers, such as breast cancer, osteosarcomas, liver cancer, and prostate cancer (Gabay et al., 2014). The expression of c-Myc is closely related to the occurrence and development of many kinds of tumors, including pancreatic cancer, but the role of c-Myc in bufalin-treated pancreatic cancer is yet to be clarified. Therefore, this study aimed to investigate the role of c-Myc in pancreatic cancer and its effect on bufalin-treated pancreatic cancer.

## Materials and Methods

### Reagents

Bufalin (98% purity as per the manufacturer, determined *via* high-performance liquid chromatography; CAS: 465-90-7, batch number: B24688-5mg) was purchased from Shanghai Yuanye Bio-Technology Co., Ltd. (Shanghai, China). The chemical structure is shown in **Figure 2A**.

### Cell Lines and Cell Culture

Human pancreatic cancer cell lines BxPC3, SW1990, and PANC-1 were purchased from iCell Bioscience Inc (Shanghai, China). HS766T and colo357 cell lines were obtained from Shanghai Jining Shiye (Shanghai, China). PCI-35 cell was purchased from Hangzhou Young Eagle Biotechnology Co., Ltd (Hangzhou China). PANC-1, HS766T, and Colo357 cells were cultured in Dulbecco’s modified Eagle medium, while SW1990, BxPC3, and PCI-35 cells were grown in RPMI-1640 medium (HyClone Laboratories Inc., Waltham, Massachusetts, USA). All medium contained 10% fetal bovine serum (FBS, Zhejiang Tianhang Biotechnology Co., Ltd. Hangzhou, China), penicillin (100 U/ml), and streptomycin (100 µg/ml). Cells were maintained at 37°C in a humidified atmosphere of 5% CO_2_.

### Cell Transfection

c-Myc siRNA and overexpression plasmid were purchased from Hangzhou Young Eagle Biotechnology Co., Ltd. The siRNA sequences were as follows: NC siRNA, forward: 5′-CGUACGCGGAAUACUUCGATT-3′; reverse: 5′-UCGAAGUAUUCCGCGUACGTT-3′; c-Myc RNA, forward: 5′-AACAGAAAUGUCCUGAGCAAUTT-3′; reverse: 5′-AUUGCUCAGGACAUUUCUGUUTT-3′. The cells were divided into blank, negative control (si-NC/pcDNA), and si-c-Myc/pcDNA-c-Myc. PANC-1 and SW1990 cell lines were used because c-Myc expression was the highest and lowest, respectively, in these cell lines. Cells (1×10^6^/well) were seeded in 6-well plates and cultured at 50%–60% confluency. Transient transfection of cells was performed using lipofectamine 2000 (Invitrogen; Thermo Fisher Scientific, Inc.) following the manufacturer’s protocol. The transfected RNA or DNA were dissolved in Opti-MEM and incubated with Lipofectamine-2000 at room temperature for 20 min to form a compound. Then, the solution was added into each well and incubated at 37°C for 48 h. c-Myc expression was detected *via* western blot and quantitative real-time polymerase chain reaction (qRT-PCR).

### qRT-PCR

Total RNA was extracted using Trizol reagent (Sangon Biotech Co., Ltd. Shanghai, China). RNA purity and concentrations were detected *via* the spectrophotometric method (Thermo Scientific, Waltham, MA, USA). Total RNA was reverse transcribed into cDNA using a cDNA Reverse Transcription kit (CoWin Biosciences, Taizhou, Jiangsu, China) according to the manufacturer’s protocol. PCR was conducted using SYBR Green qPCR kit (CoWin Biosciences) and followed by amplification using LightCycler^®^ 96 Real-Time PCR System (Roche Molecular Systems, Inc. Basle, Switzerland) at 95°C for 10 min, 95°C for 15 s, and 60°C for 60 s (40 cycles). The relative mRNA expression was calculated using the 2 ^−ΔΔCt^ method and normalized against β-actin. The primer sequences were: c-Myc, forward: 5′-GGCTCCTGGCAAAAGGTCA-3′; reverse: 5′-CTGCGTAGTTGTGCTGATGT-3′; β-actin, forward: 5′-TGAGCGCGGCTACAGCTT-3′; reverse: 5′-TCCTTAATGTCACGCACGATTT-3′.

### Protein Extraction and Western Blot Analysis

The cells and tissue specimens were lysed with radioimmunoprecipitation assay buffer (Beyotime Institute of Biotechnology, Shanghai, China) containing phenylmethanesulfonyl fluoride (Beyotime) and a protease inhibitor (CoWin Biosciences). The protein concentration was determined using a bicinchoninic acid protein assay kit (Beijing Solarbio Science and Technology Co., Ltd. Beijing, China). Each sample with equal amounts of protein were separated using 10% sodium dodecyl sulfate polyacrylamide gel electrophoresis. After running 1–2 h under 120V, the protein was transferred onto a polyvinylidene difluoride membrane. Next, the membranes were blocked with 5% not-fat milk for 1 h at room temperature and incubated with primary antibodies c-Myc, HIF-1α, CXCR4, SDF-1, vimentin, E-cadherin, and β-actin overnight at 4°C (Affinity Biosciences). Subsequently, the membranes were washed 3 times with tris-buffered saline and Tween-20 and incubated with a horseradish peroxidase-conjugated goat anti-rabbit or anti-mouse secondary antibody (Cell Signaling Technology, Beverly, MA, USA). Finally, the membranes were treated with a chemiluminescence reagent and exposed to X-ray. The band intensities were quantified using image J software (National Institutes of Health, Bethesda, MD, USA). Relative protein expression was quantified using control protein β-actin.

### Cell Viability Assay

Cell viability was measured using Cell Counting Kit-8 (CCK-8; MedChemExpress LLC, USA). Cells were seeded into 96-well plates and incubated for 24 h. Bufalin was diluted to a final concentration of 80 nmol/L in dimethyl sulfoxide (DMSO). Cells treated with only DMSO (0.1%) served as control. The experiment was carried out according to the above groups. After 24-h treatment, 10 μl CCK-8 solution was added to each well, and the cells were incubated for 0.5–2 h at 37°C. The absorbance was measured at 450 nm using a microplate reader (SpectiaMax, Molecular Devices, USA). All of the experiments were repeated at least three times. The cell viability percentage was calculated by comparison of the treatment OD to that of the control.

### Flow Cytometry and Cell Cycle Analysis

Cells were incubated with DMSO and bufalin as the above groups for 24 h. Then, the cells were collected and fixed with cold 70% ethanol at -20°C overnight. After centrifugation, the fixed cells were washed twice with phosphate-buffered saline (PBS) resuspended with PBS containing 100 μg/ml RNase and 5 μg/ml propidium iodide, and incubated for 30 min at room temperature in the dark. Then, the cells were analyzed using FACS Accuri C6 flow cytometer (BD Biosciences, Franklin Lakes, NJ, USA), with an analysis software determining the percentage of cells during the different phases of the cell cycle.

### Cell Apoptosis Analysis

Cell apoptosis was analyzed using Annexin V-FITC/PI Apoptosis Detection Kit (CoWin Biosciences) according to the manufacturer’s instruction. Briefly, after treatment with DMSO and bufalin (24 h), the cells were collected and washed twice with PBS, and resuspended with annexin V binding buffer. Then, cells were stained with 5 μl annexin V/FITC and 10 μl 20 μg/ml propidium iodide (PI) for 15 min in the dark at room temperature. The stained cells were analyzed using FACS Accuri C6 (BD Biosciences).

### Transwell Assay

Cell invasion assay was performed using Transwell chambers (Corning, NY, USA) coated with Matrigel (BD Biosciences). Cells were treated with bufalin as above groups for 24 h. Transwell chambers were placed in the 24-well plates. Cells (5×10^4^) in serum-free medium were placed into the upper chamber coated with Matrigel, and medium containing 10% FBS was added to the lower chamber. After 24 hours of incubation at 37°C, the non-invasive cells were washed, and cells that had invaded through the membrane were fixed and stained with methanol and crystal violet. Then, the cells were counted and imaged using an inverted microscope (Nikon, Tokyo, Japan).

### Wound Healing Assay

Cell migration was measured *via* cell scratch test. Cells were seeded on 6-well plates and cultured to a confluent monolayer. Then, the scratches were performed using a pipette tip (200 µl), and the scraped cells were washed thrice with PBS. The cells were treated according to the above groups and continued to be cultured and replaced with serum-free medium. The scratch distance was detected and photographed after 0 h and 24 h. The percentage of cell migration was calculated in the pictures using image J software (National Institutes of Health).

### Animal Models and Experiments

We purchased 4-week-old male athymic BALB/c nude mice from the SLAC Laboratory Animals Co. Ltd. (Shanghai, China) and maintained in the Zhejiang Chinese Medical University Laboratory Animal Research Center. SW1990/PANC-1-1 cells (1×10^6^) in 0.2 ml PBS were injected in the 24 mice and allowed to grow for 7 days to reach a tumor size of more than 50 mm^3^. Then, the mice injected with different cells were randomly divided into the control group, the positive group (cisplatin, diaminedichloroplatinum [DDP]), and the bufalin group, with eight mice per group. Mice in the control group were injected intra-peritoneally (i.p.) with normal saline, while those in the treatment group were injected i.p. with DDP and bufalin at a dosage of 2 mg/kg per day for 14 days. The tumor volume was measured twice weekly. At the end of the experiments, the mice were sacrificed, and the tumor were dissected to measure the volume ((length × width^2^) × 0.5). Then, the tumor tissues were cut into slices for further pathological experiment.

The animal protocol experiment was approved by the Ethics Committee of Zhejiang Chinese Medical University. All applicable international, national, and/or institutional guidelines for the care and use of animals were followed.

### Hematoxylin-Eosin (HE) Staining

HE staining was used to evaluate the effect of bufalin on the pathological changes of pancreatic cancer in mice. Deparaffinized and rehydrated tumor tissue sections were stained with hematoxylin (Wuhan Servicebio Technology Co., Ltd. Wuhan, China) for 5 min and rinsed with running water. Then, the sections were differentiated using hydrochloric acid ethanol for 30 s and socked in tap water for 15 min. Next, the sections were stained with eosin (Zhuhai Beso Biotechnology Co., Ltd. Zhuhai, Guangdong, China) for 2 min, rinsed with water, and dehydrated for transparency. The pathological changes were examined and photographed using an optical microscope.

### Terminal Deoxynucleotidyl Transferase-Mediated dUTP Nick-End Labeling (TUNEL) Assay

The TUNEL assay was used to evaluate cell apoptosis. Briefly, after xylene dewaxing and gradient ethanol dehydration, the TUNEL assay was performed according to the instructions in the apoptosis detection kit (Beyotime). Apoptotic cells are stained brown. The result was photographed using an optical microscope and analyzed by image J software.

### Statistical Analysis

Data were expressed as mean ± standard deviation. The results were analyzed using student’s t test or one-way ANOVA. All statistical analyses were carried out using SPSS 19.0 software (SPSS Inc., Chicago, IL, USA), and results were presented with Graph Prism 8.0 software (GraphPad Software, Inc., La Jolla, CA, USA). *P* < 0.05 was considered as statistically significant.

## Results

### Construction of the Cell Lines With Different c-Myc Expression

**Figure 1:**
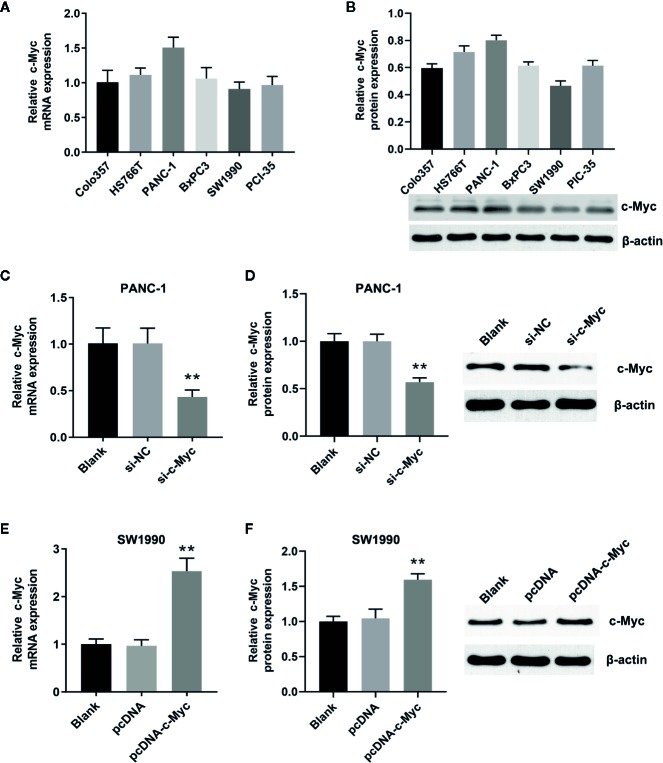
Construction of the cell lines with different c-Myc expression. The expression of c-Myc in human pancreatic cancer cells (Colo357, HS766T, PANC-1, BxPC3, SW1990, PIC-35) was detected *via* **(A)** Quantitative real-time polymerase chain reaction (qRT-PCR) and **(B)** western blot (n = 3). The expression of c-Myc in PANC-1 cells transfected with si-c-Myc or siRNA negative control was detected *via* **(C)** qRT-PCR and **(D)** western blot (^**^ *p* < 0.01 vs control, n = 3). The expression of c-Myc in SW1990 cells transfected with pcDNA-c-Myc or empty vector pcDNA was detected *via* **(E)** qRT-PCR and **(F)** western blot (^**^ *p* < 0.01 vs control, n = 3).

qRT-PCR and western blot analysis showed that c-Myc was expressed in Colo357, HS76T, PANC-1, BxPC3, SW1990, and PCI-35 pancreatic cells (**Figures 1A, B**), with the highest and lowest expression in PANC-1 and SW1990 cells, respectively. Thus, PANC-1 and SW1990 cells were selected for further experiments. To evaluate the role of c-Myc in pancreatic cancer cells, siRNA targeting c-Myc (si-c-Myc) and siRNA negative control (si-NC) were transfected into PANC-1 cells, and the c-Myc overexpression vector (pcDNA-c-Myc) and empty vector (pcDNA) were transfected into SW1990 cells. Compared with the control, c-Myc expression in PANC-1 cells was reduced significantly after transfection with si-c-Myc (*P* < 0.01; **Figures 1C, D**). Similarly, c-Myc expression in SW1990 cells transfected with pcDNA-c-Myc was significantly increased, compared with the control (*P* < 0.01; **Figures 1E, F**). The results suggested that the cell line model with different c-Myc expression was successfully constructed.

### Downregulation of c-Myc Expression Enhanced the Inhibition Effect of Bufalin on Cell Proliferation in Pancreatic Cancer Cells

**Figure 2:**
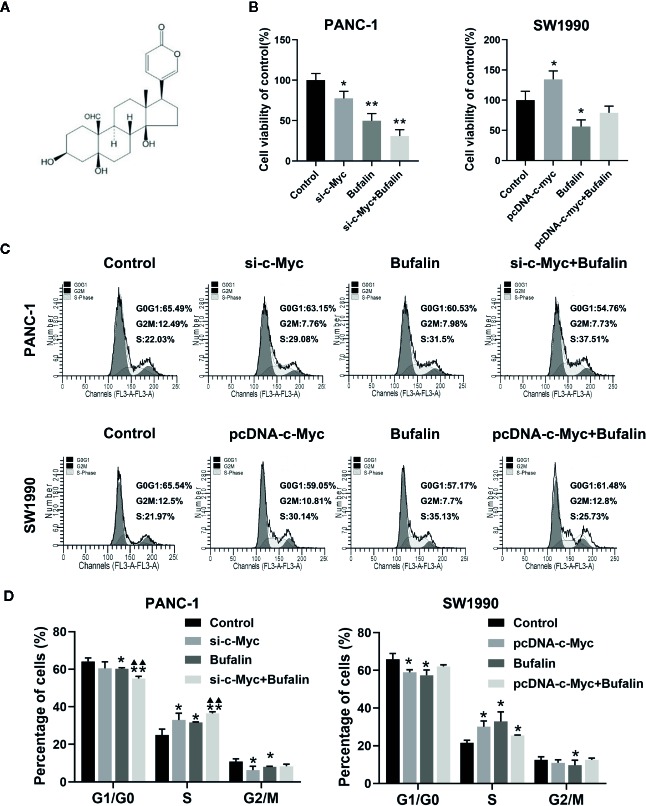
Downregulation of c-Myc enhanced the inhibition effect of bufalin on cell proliferation and cell cycle in pancreatic cancer cells. PANC-1 and SW1990 cells were transfected with si-c-Myc and pcDNA-c-Myc, respectively. Cells were treated with dimethyl sulfoxide (DMSO) or bufalin (80 nmol/L) for 24 h. **(A)** The structure of bufalin. **(B)** The viability of PANC-1 and SW1990 cells were detected *via* CCK-8 assay (^*^ *p* < 0.05, ^**^ *p* < 0.01 vs control, n = 3). **(C)** Cell cycle distribution was analyzed *via* flow cytometry. **(D)** Statistical histograms of cells in the G1/G0, S, and G2/M phases of the cell cycle (^*^ *p* < 0.05, ^**^ *p* < 0.01 vs control, ^▲▲^ *p* < 0.01 vs bufalin treatment group, n = 3).

As shown in **Figure 2B**, bufalin inhibits the cell viability of PANC-1 and SW1990 cells, compared with the control. To determine the underlying role of c-Myc in bufalin-mediated inhibition of cell proliferation, we evaluated the effects of bufalin on pancreatic cancer cells after knockdown and overexpression of c-Myc. The results of CCK-8 assay revealed that decreased expression of c-Myc could inhibit PANC-1 cell growth, while its overexpression could increase cell proliferation, when compared with the control (*P* < 0.05; **Figure 2B**). This result indicated that c-Myc expression is critical for the proliferation of pancreatic cancer cells.

### Downregulation of c-Myc Expression Enhanced the Effect of Bufalin on Cell Cycle Arrest in Pancreatic Cancer Cells

In our previous study, we have reported that bufalin inhibits pancreatic cancer cell growth by inducing cell cycle arrest at the S phase (Liu et al., 2016). Here, we analyzed the effect of c-Myc on the cell cycle in bufalin-treated pancreatic cancer cells. Flow cytometry analysis showed that compared with the bufalin only treatment group without c-Myc knockdown, cell cycle arrest at the S phase was enhanced in the bufalin-incubated group with c-Myc knockdown (*P* < 0.01; **Figures 2C, D**). The results revealed that downregulation of c-Myc could enhance the effect of bufalin on arresting cell cycle at S phase.

### Downregulation of c-Myc Enhanced Bufalin-Induced Apoptosis of Pancreatic Cancer Cells

**Figure 3:**
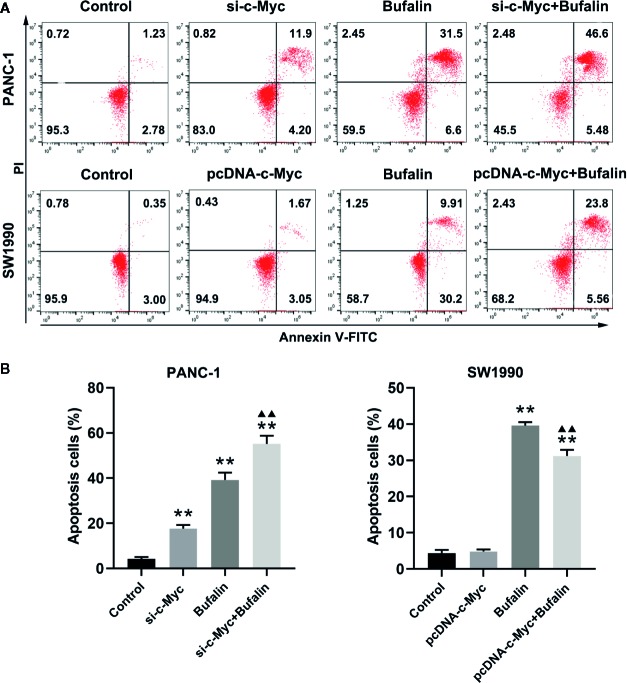
Downregulation of c-Myc enhanced bufalin-induced apoptosis of pancreatic cancer cells. Cells were treated with dimethyl sulfoxide (DMSO) or bufalin (80 nmol/L) for 24 h. **(A)** The apoptosis rate of PANC-1 and SW1990 cells was measured *via* flow cytometry. **(B)** Statistical histograms of the apoptosis rate of PANC-1 and SW1990 cells (^**^ *p* < 0.01 vs control, ^▲▲^ *p* < 0.01 vs bufalin treatment group, n = 3).

As shown in **Figures 3A, B**, bufalin increased cell apoptosis in PANC-1 and SW1990 pancreatic cells. Our analysis of the influence of c-Myc on pancreatic cell apoptosis and its role in bufalin-induced cell apoptosis showed that decreased c-Myc expression could increase apoptosis in PANC-1 pancreatic cells (*P* < 0.01). Furthermore, in PANC-1 pancreatic cells, bufalin-induced apoptosis was significantly enhanced in the bufalin-incubated group with c-Myc knockdown compared with the bufalin treatment only group without c-Myc knockdown (*P* < 0.01). Similarly, bufalin-induced SW1990 cell apoptosis was significantly decreased in the bufalin-incubated group with c-Myc overexpression group compared with the bufalin only treatment group without c-Myc overexpression (*P* < 0.01). These results indicated that c-Myc inhibits pancreatic cell apoptosis, and its downregulation enhanced bufalin-induced apoptosis of pancreatic cancer cells.

### Downregulation of c-Myc Enhanced Bufalin-Induced Suppression of Pancreatic Cancer Cell Invasion and Migration

**Figure 4:**
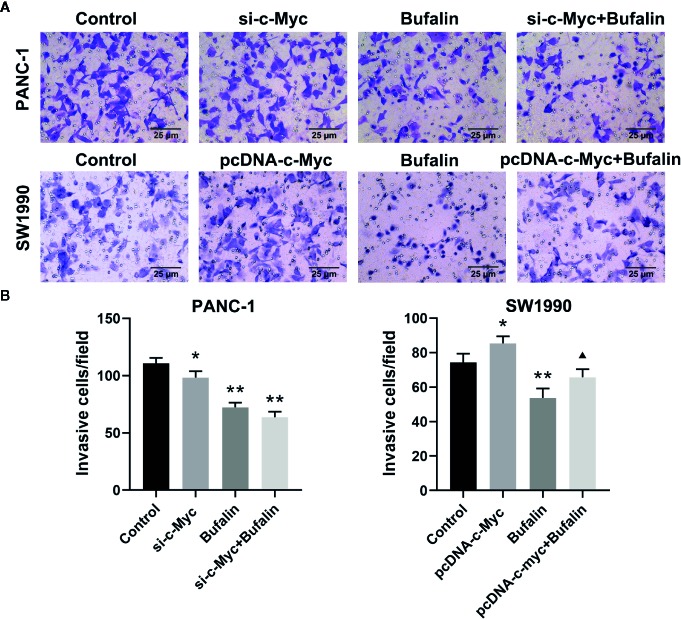
Downregulation of c-Myc enhanced the suppression effect of bufalin on pancreatic cancer cell invasion. Cells were treated with dimethyl sulfoxide (DMSO) or bufalin (80 nmol/L) for 24 h. **(A)** The invasive capacity of PANC-1 and SW1990 cells was measured *via* Transwell assay (magnification, ×200). **(B)** Statistical histograms of the invasive cells per field (^*^ *p* < 0.05, ^**^ *p* < 0.01 vs control, ^▲^ *p* < 0.05 vs bufalin treatment group, n = 3).

**Figure 5:**
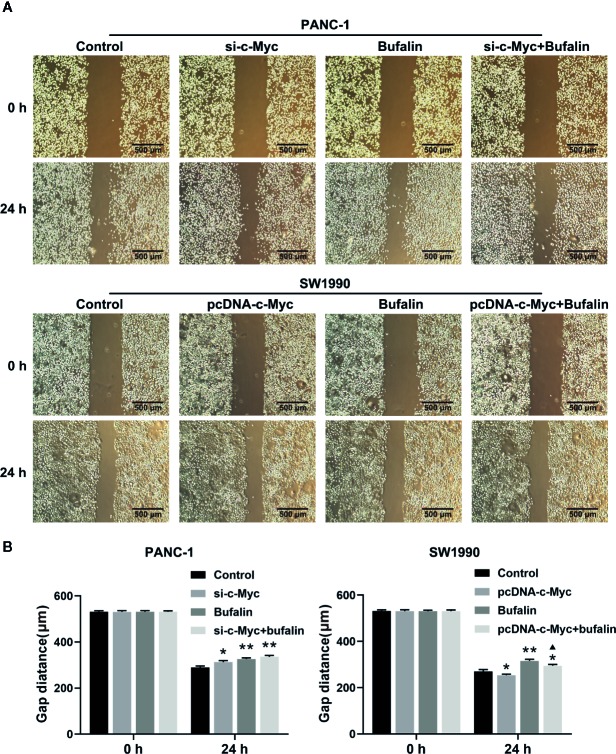
Downregulation of c-Myc enhanced the suppression effect of bufalin on pancreatic cancer cell migration. The scratches were performed after the cells were cultured in a confluent monolayer. Cells were treated with dimethyl sulfoxide (DMSO) or bufalin (80 nmol/L). The scratch distance was detected and photographed after 0 h and 24 h. **(A)** The migration capacity of PANC-1 and SW1990 cells was measured *via* wound healing assay (magnification, ×40). **(B)** Statistical histograms of the gap distances (^*^ *p* < 0.05, ^**^ *p* < 0.01 vs control, ^▲^ *p* < 0.05 vs bufalin treatment group, n = 3).

**Figure 6:**
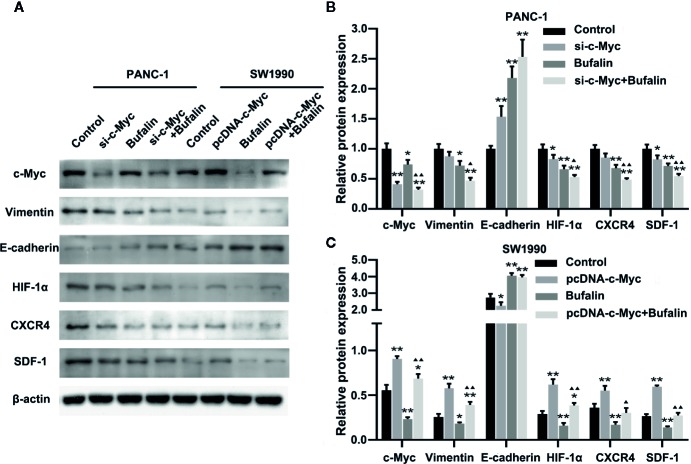
Downregulation of c-Myc enhanced the antitumor effect of bufalin in pancreatic cancer cells through the HIF-1α/SDF-1/CXCR4 pathway. **(A)** The protein expression of c-Myc, vimentin, E-cadherin, HIF-1α, CXCR4, and SDF-1 in PANC-1 and SW1990 pancreatic cancer cells under different treatments was detected *via* western blot. **(B)** Quantification results of protein expressions of c-Myc, vimentin, E-cadherin, HIF-1α, CXCR4, and SDF-1 in PANC-1 pancreatic cancer cells. **(C)** Quantification results of protein expression of c-Myc, vimentin, E-cadherin, HIF-1α, CXCR4, and SDF-1 in SW1990 pancreatic cancer cells (^*^ *p* < 0.05, ^**^ *p* < 0.01 vs control, ^▲^ *p* < 0.05, ^▲▲^ *p* < 0.01 vs bufalin treatment group, n = 3).

The results of Transwell and wound healing assays showed that PANC-1 and SW1990 cell invasion and migration were significantly inhibited in the bufalin treatment group compared with the control group (**Figures 4A, B**; **Figures 5A, B**). The Transwell assay also demonstrated that c-Myc overexpression promoted SW1990 cell invasion compared with the control group (*P* < 0.05; **Figures 4A, B**), and c-Myc overexpression increased the number of invasive cells after bufalin incubation compared with bufalin only treatment (*P* < 0.05; **Figures 4A, B**). Wound healing assay revealed that the migration distance of c-Myc overexpression SW1990 cells was longer compared with that in the control group (*P* < 0.05; **Figures 5A, B**), and the distance was decreased after incubation with bufalin for 24 h compared with bufalin treatment only (*P* < 0.05; **Figures 5A, B**). Western blot assay to evaluate the effect of bufalin and c-Myc expression on epithelial-mesenchymal transition (EMT) markers, including vimentin and E-cadherin protein, showed that bufalin increased the expression of E-cadherin and decreased the expression of vimentin in pancreatic cells, indicating that bufalin could inhibit EMT, and downregulation of c-Myc expression enhanced this effect (**Figures 6A–C**). By contrast, c-Myc overexpression induced the opposite effect (**Figures 6A, C**). These results indicated that c-Myc promoted the invasion and migration of pancreatic cells, and c-Myc knockdown could enhance the effect of bufalin on EMT.

### Downregulation of c-Myc Regulated the HIF-1α/SDF-1/CXCR4 Pathway to Enhance the Antitumor Effect of Bufalin in Pancreatic Cancer Cells

Previous studies have shown SDF-1/CXCR4 signaling induces pancreatic cancer cell invasion and EMT (Li et al., 2012). Thus, we then investigated the molecular mechanism by which c-Myc enhances the effect of bufalin in pancreatic cancer cells. We examined the role of HIF-1α, CXCR4, and SDF-1 protein in c-Myc regulation in pancreatic cancer cells. As shown in **Figures 6A, B**, c-Myc knockdown in PANC-1 cells resulted in significantly decreased expression of HIF-1α, CXCR4, and SDF-1 compared with the control (*P* < 0.05), and the effect was increased when combined with bufalin treatment (*P* < 0.05 or *P* < 0.01). These results were further verified *via* c-Myc overexpression (**Figures 6A, C**). Collectively, these results suggested that c-Myc regulated the HIF-1α/SDF-1/CXCR4 pathway in pancreatic cancer cells to enhance the antitumor effect of bufalin.

### Bufalin Inhibited Tumor Growth in Nude Mice Through the HIF-1α/SDF-1/CXCR4 Pathway

**Figure 7:**
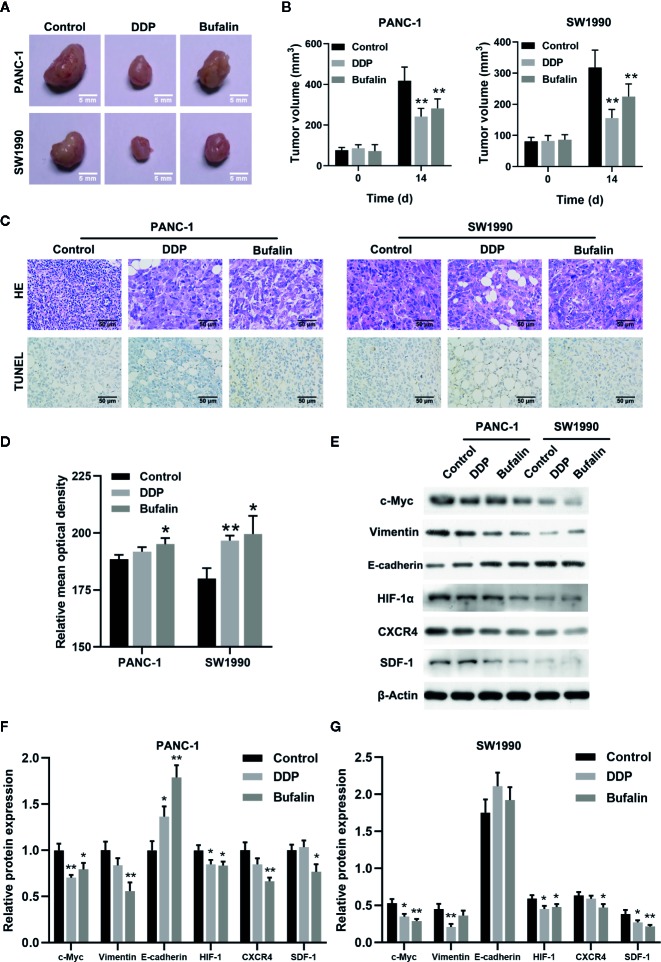
Bufalin inhibited tumor growth in nude mice by regulating the HIF-1α/SDF-1/CXCR4 pathway. The nude mice were injected with PANC-1 and SW1990 cells, respectively, and grew for 7 days. Xenograft tumor masses were harvested after 14 days following administration of bufalin or diaminedichloroplatinum (DDP), with the group treated with normal saline set as control. **(A)** The tumor size was assessed via caliper measurement. **(B)** Statistical histograms showing tumor size (**p < 0.01 vs control, n = 3). **(C)** The tumor tissues were cut into slices and subjected to 2.12 hematoxylin-eosin (HE) and TUNEL assays. HE staining showed the pathological changes of the pancreatic cells in mice (magnification, ×400). TUNEL assay showed the apoptosis of the pancreatic cells in mice (magnification, ×400). **(D)** Statistical histograms of the apoptotic cells. Image J was used to analyze the relative IOD value of apoptotic cells. (^*^ *p* < 0.05, ^**^ *p* < 0.01 vs control, n = 3). **(E)** The protein expression of c-Myc, vimentin, E-cadherin, HIF-1α, CXCR4, and SDF-1 in PANC-1 and SW1990 pancreatic cancer tissues in the control, DDP, and bufalin treatment groups was detected *via* western blot. **(F)** Quantification results of protein expression of c-Myc, vimentin, E-cadherin, HIF-1α, CXCR4, and SDF-1 in PANC-1 pancreatic cancer tissues. **(G)** Quantification results of protein expression of c-Myc, vimentin, E-cadherin, HIF-1α, CXCR4, and SDF-1 in SW1990 pancreatic cancer tissues (^*^ *p* < 0.05, ^**^ *p* < 0.01 vs control, n = 3).

As shown previously, bufalin inhibits the proliferation, invasion, and migration of pancreatic cancer cells *in vitro*. Thus, we validated the effect of bufalin on the cancer cell growth through tumor-forming experiments in nude mice. Tumor volume was significantly inhibited in the bufalin treatment group compared with the control group (*P* < 0.01; **Figures 7A, B**). HE staining showed that the tumor cells in the control group displayed active growth and obvious mitotic phase, while the tumor tissue of bufalin treatment groups showed large areas of necrosis and inflammatory exudation compared with the control group (**Figure 7C**). TUNEL staining assays revealed that the number of apoptotic cells increased in the DDP and bufalin group compared with the control group (**Figures 7C, D**). Western blot analysis of the expression of EMT-associated proteins and HIF-1α/SDF-1/CXCR4 of pancreatic cancer tissues revealed that bufalin significantly downregulated vimentin but upregulated E-cadherin in PANC-1 cells. Further, bufalin significantly inhibited the expression of c-Myc and HIF-1α/SDF-1/CXCR4 (*P* < 0.05 or *P* < 0.01; **Figures 7E–G**), which was consistent with the results *in vitro*. These results support that bufalin inhibits the growth of pancreatic cancer cells by downregulating the HIF-1α/SDF-1/CXCR4 pathway, which was regulated by c-Myc.

## Discussion

Pancreatic cancer is one of the most lethal cancers worldwide because it is highly invasive and metastatic. The GLOBOCAN 2012 report shows that pancreatic cancer causes more than 331,000 deaths annually (Ferlay et al., 2015). Surgical resection is currently the only curative treatment modality for pancreatic cancer, but most patients are ineligible as they already have metastasis during initial diagnosis. Further, the tumor resistance to chemotherapy and limited knowledge of the underlying mechanisms that cause the malignance of this cancer make the treatment of pancreatic patients a challenge. Bufalin, as a traditional Chinese medicine, has been demonstrated to inhibit cancer cell proliferation by inducing apoptosis and cell cycle arrest in many cancers (Takai et al., 2012; Yin et al., 2012) and also few works have been reported that bufalin could suppress the invasion and migration of cancer cells (Qiu et al., 2013; Wang et al., 2018). In our previous study, we showed that bufalin inhibits the proliferation of pancreatic cancer cells by inducing apoptosis and cell cycle arrest at S phase and the possible mechanisms for this effect might be bufalin inhibits the transcriptional activity and decreases the expression of c-Myc. Therefore, we performed the further mechanism study in present study, and the results showed that downregulation of c-Myc enhanced the antitumor activity of bufalin on pancreatic cancer cells by suppressing the HIF-1α/SDF-1/CXCR4 pathway.

c-Myc, as a transcriptional factor, activates many genes that are involved in cellular processes, including cell growth, differentiation, apoptosis, metabolism, angiogenesis and transformation (Li et al., 2012). The role of c-Myc in tumor growth and inhibition of tumor progression are being studied extensively. Some studies have shown elevated expression of c-MYC oncogene or its protein product c-Myc in pancreatic cancer (Gabay et al., 2014; La Rosa et al., 2018). Other studies showed that c-Myc was deregulated in pancreatic cancer, and only cytoplasmic c-Myc expression was an independent prognostic biomarker of pancreatic cancer (He et al., 2014; Kalkat et al., 2017). In addition, c-Myc overexpression was considered to be closely related to the tumor-node-metastasis stage, tumor size, and poor prognosis of pancreatic cancer (He et al., 2015). He et al. reported that expression of c-Myc and Fas played an important role in perineural invasion of pancreatic cancer (He et al., 2012). Thus, in this case, we also investigated the role of c-Myc in pancreatic cancer cells. Our results showed that c-Myc knockdown could inhibit the proliferation, invasion, and migration of pancreatic cancer cells, and induce EMT with the upregulation of E-cadherin and downregulation of vimentin. EMT is simply defined as the phenotypic transition from an epithelial to a mesenchymal state with increased cell migration and invasion. Several studies have demonstrated that the invasiveness of pancreatic cancer correlates with the EMT (Wang et al., 2017). Further, increasing evidence has shown that the EMT played a key role in tumor metastasis (Rhim et al., 2012; Zheng et al., 2015). c-Myc overexpression caused the opposite results. These results indicate that c-Myc plays an important role in the process of pancreatic tumorigenesis and invasion. Therefore, c-Myc may be considered a useful biomarker for metastasis of pancreatic cancer.

Bufalin inhibits metastasis in different cancer types, including liver and lung cancer (Wu et al., 2015; Wang et al., 2016). An important strategy in cancer treatment is to prevent or reduce the metastasis of cancer cells. However, few studies have investigated the mechanism of bufalin-inhibited migration and invasion in human pancreatic cancer cells. Therefore, we investigated the effect of bufalin on invasion, migration, and EMT-related protein in pancreatic cancer cells along with c-Myc siRNA and c-Myc pcDNA3.1. Our results demonstrated that c-Myc downregulation enhanced the inhibition effect of bufalin on the invasion and migration in pancreatic cancer cells. Furthermore, bufalin significantly downregulated the mesenchymal marker vimentin and upregulated the epithelial marker E-cadherin *in vivo* and *in vitro*. Moreover, c-Myc downregulation improved the inhibition effect of bufalin on EMT. These results provide evidence on the feasibility of combination of anti-c-Myc and bufalin in the treatment of pancreatic cancer.

HIF-1α, a signal transcription factor, plays an important role in tumorigenesis, development, invasion, metastasis, and apoptosis (Semenza, 2003). It was reported that bufalin suppressed hepatocellular carcinoma invasion and metastasis by targeting HIF-1α (Wang et al., 2016). SDF-1 is a member of the CXC chemokine family and is a ligand for CXCR4 (Koshiba et al., 2000). Increasing evidence showed that SDF-1/CXCR4 signaling was correlated to cancer (Gao et al., 2010). Further, previous studies have demonstrated that CXCR4 was highly expressed in pancreatic cancer, and the elevated levels of SDF-1 and CXCR4 were associated with poor prognosis (Wehler et al., 2006; Liang et al., 2010). Recent studies have verified that SDF-1/CXCR4 signaling could induce pancreatic cancer cell invasion and EMT *in vitro* (Li et al., 2012). Our results revealed that bufalin inhibited the expression of HIF-1α/SDF-1/CXCR4 *in vitro* and *in vivo*. Further, c-Myc knockdown decreased the expression of HIF-1α/SDF-1/CXCR4 and enhanced the antitumor effect of bufalin in pancreatic cancer cells. Conversely, c-Myc overexpression upregulated the expression of HIF-1α/SDF-1/CXCR4 and impaired the effect of bufalin. These results suggested that the HIF-1α/SDF-1/CXCR4 signaling pathway might be a promising therapeutic target to prevent pancreatic cancer progression.

In conclusion, our study showed that c-Myc could promote the development of pancreatic cancer, and c-Myc downregulation could synergistically enhance the antitumor activity of bufalin by downregulating the HIF-1α/SDF-1/CXCR4 pathway. This study provides a theoretical support for using bufalin combined with anti-c-Myc in the treatment of pancreatic cancer and expanding the application of bufalin in clinical practice. Moreover, the HIF-1α/SDF-1/CXCR4 signaling pathway may be a potential therapeutic target for pancreatic cancer.

## Data Availability Statement

The datasets used and/or analyzed during the current study are available from the corresponding authors upon reasonable request.

## Ethics Statement

The animal protocol experiment was approved by the Ethics Committee of Zhejiang Chinese Medical University.

## Author Contributions

QS and JW designed the experiments. XL and YZ wrote the manuscript. YZ and BX performed the experiments. JP analyzed the data. All authors read and approved the final manuscript.

## Funding

This work was supported by the Natural Science Foundation of Zhejiang Province (grant nos. LY18H280006 and LQ17H030006), National Natural Science Foundation of China (grant no.81873047) and the Youth Scientific Research Innovation Fund of Zhejiang Chinese Medical University (grant no. KC201938)

## Conflict of Interest

The authors declare that the research was conducted in the absence of any commercial or financial relationships that could be construed as a potential conflict of interest.
